# LOV-based optogenetic devices: light-driven modules to impart photoregulated control of cellular signaling

**DOI:** 10.3389/fmolb.2015.00018

**Published:** 2015-05-12

**Authors:** Ashutosh Pudasaini, Kaley K. El-Arab, Brian D. Zoltowski

**Affiliations:** Department of Chemistry, Center for Drug Discovery, Design and Delivery at Dedman College, Southern Methodist UniversityDallas, TX, USA

**Keywords:** LOV domain, optogenetics, protein engineering, photobiology, photosensors

## Abstract

The Light-Oxygen-Voltage domain family of proteins is widespread in biology where they impart sensory responses to signal transduction domains. The small, light responsive LOV modules offer a novel platform for the construction of optogenetic tools. Currently, the design and implementation of these devices is partially hindered by a lack of understanding of how light drives allosteric changes in protein conformation to activate diverse signal transduction domains. Further, divergent photocycle properties amongst LOV family members complicate construction of highly sensitive devices with fast on/off kinetics. In the present review we discuss the history of LOV domain research with primary emphasis on tuning LOV domain chemistry and signal transduction to allow for improved optogenetic tools.

## Introduction

Over the past 10 years, advancements in our understanding of photoactivated proteins have enabled genetic control of cellular events through light. These optogenetic approaches allow researchers to dictate biological signaling with exquisite spatial and temporal precision. The ability to remotely and non-invasively trigger signal transduction has led to unparalleled breakthroughs in neuroscience, cardiology and cell biology (Moglich and Moffat, 2010; Boyden, 2011; Fenno et al., 2011; Deisseroth, 2012). Whereas, initially most research focused on the use of light-controlled opsins to affect neurobiology, more recent research has employed a host of photoactivatable proteins from the Light-Oxygen-Voltage (LOV), Cryptochrome (CRYs), Blue-light-using FAD (BLUF), Phytochrome (PHY), and UVR8 families of proteins (Moglich and Moffat, 2010; Fenno et al., 2011; Christie et al., 2012a). A central goal of these efforts has been to identify a protein module that can, in an efficient and robust manner, be coupled to any signaling domain to elicit photoregulated control. Despite a wide range of functional devices that have been developed, several key limitations exist in developing the ideal optogenetic tool. Given the breadth of the field and diverse reviews in the subject matter, the present review will focus on LOV-based optogenetic devices. Specific focus will be on existing tools, their limitations, and current efforts to improve them for widespread usage in cell biology and medicine.

LOV domains were first identified as the photoreactive module regulating plant phototropism (Huala et al., 1997; Salomon et al., 2000). Since their initial discovery, they have been found in bacterial, algal, fungal and plant species, where they impart blue-light sensitivity to myriad signal transduction domains (Crosson et al., 2003). Structurally, LOV domains are a subclass to the wider Period-ARNT-Singleminded (PAS) domain family that is distinguished by the presence of a flavin (FMN, FAD, or riboflavin) cofactor and the presence of a consensus GXNCRFLQ motif (Taylor and Zhulin, 1999; Zoltowski and Gardner, 2011).

The LOV module is defined by a core domain of ~110 amino acids forms a PAS fold composed of a central 5-stranded antiparallel β-sheet and a helical face that bind the photoreactive flavin (Zoltowski and Gardner, 2011). Current research indicates that in nearly all cases, the core domain signals to effector elements through highly variable N-terminal (Ncap) or C-terminal (Ccap) extensions to the LOV core (Halavaty and Moffat, 2007; Zoltowski et al., 2007; Zoltowski and Crane, 2008; Nash et al., 2011; Diensthuber et al., 2013; Lokhandwala et al., 2015). These extensions are typically helical and couple LOV-photochemistry to allosteric control of effector domains. In optogenetic devices allosteric regulation of effector elements has been harnessed through three general methods that are detailed further below: (1) Light-driven protein-protein interaction modules that drive transcription or cellular localization (Strickland et al., 2008; Yazawa et al., 2009; Lungu et al., 2012; Polstein and Gersbach, 2012; Chen et al., 2013; Motta-Mena et al., 2014). (2) Light-driven activators of signaling (e.g., histidine kinases, phosphodiesterases, cell mobility) (Wu et al., 2009; Ohlendorf et al., 2012; Grusch et al., 2014; Yi et al., 2014; Yin and Wu, 2015) and (3) Fluorescent reporter molecules (Chapman et al., 2008; Mukherjee and Schroeder, 2015). Currently, these devices are still limited in the degree of activation, residual dark state function and non-ideal photochemical cycles.

**Figure 1:**
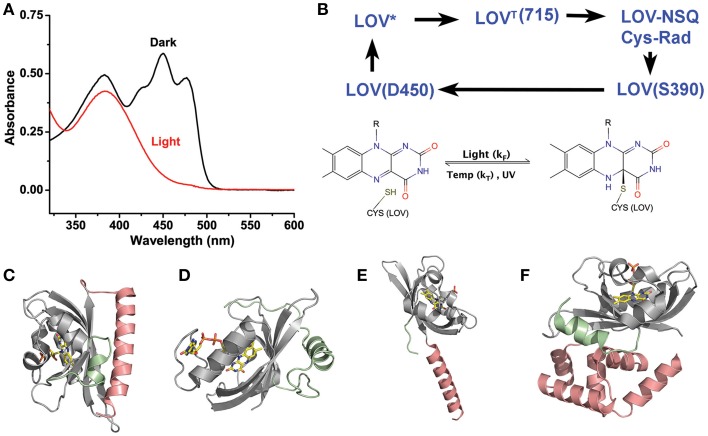
**LOV chemistry and structure. (A)** Typical photocycle spectra of LOV containing proteins. Dark state proteins (black) demonstrate spectra consistent with oxidized flavin. Light activation (red) bleaches the 450 nm absorbing bands leaving a single 390 nm peak indicative of a C4a adduct. **(B)** LOV photocycles are characterized by a ground state oxidize flavin that form a flavin-cysteine C4a adduct following blue-light treatment. Adduct formation proceeds through an excited singlet state (LOV^*^) that rapidly forms a Triplet species (LOV^T^). The triplet abstracts an electron from C38 generating a radical pair. Radical recombination forms the C4a adduct (LOV390). The adduct decays to the ground state by either thermal decay (k_T_) or UV-scission. **(C–F)** Structures of LOV proteins involved in optogenetic tools, AsLOV2 **(C)**, VVD **(D)**, YtvA **(E)**, and EL222 **(F)**. The LOV core is depicted in gray, with associated N-terminal caps (green) and C-terminal caps (salmon).

Herein we focus on five platforms that are commonly exploited as optogenetic devices; these are the LOV2 domain of *Avena sativa* phototropin 1 (AsLOV2), a fungal circadian clock photoreceptor Vivid (VVD), a *Bacillus subtilis* stress response protein (YtvA), a FLAVIN-BINDING, KELCH REPEAT, F-BOX 1 essential to plant flowering (FKF1), and a 222 amino acid LOV-transcription factor present in *Erythrobacter litoralis* (EL222) (Figure 1). These are summarized in Table 1. However, before going into detailed accounts of signal transduction mechanisms in existing LOV-based optogenetic tools, we briefly outline the current state of LOV photochemistry.

**Table 1:** **Selected LOV-based optogenetic tools**.

**LOV-system**	**Effectors**	**Fold activation**	**Purpose**	**Lifetime (s)**	**References**
**AsLOV2**	**Phototropism**			**2−4300 s (*WT* = 55-81)**	**a**
LINuSs1	NLS	3–7	Nuclear localization^20^	240 s (4 min)	Yazawa et al., 2009
LOV-TAP	TrpR	6, 70	DNA-binding/Tryptophan repressor	NA	Strickland et al., 2008; Wu et al., 2011
LOV-Rac	Rac1 (GTPase)	10	Control actin cytoskeletal dynamics	43 s	Wu et al., 2009
Tulips	ePDZ	2–49	Peptide caging		Strickland et al., 2012
LOV-TetR	TetR	NA	Tetracycline/DNA-binding	30 s	Moon et al., 2014
**YtvA**	**Stress**			**72−16000 (*WT* = 6240)**	**a**
Dusk/Dawn	FixL/FixJ	460	Transcription	NA	Ohlendorf et al., 2012
YF1	FixL	68	Kinase activity	~5900	Moglich et al., 2009
TetR	TetR	NA	Tetracycline/DNA-binding	2700	(100)
**EL222**	**Transcription**			**2.7−2000 (*WT* = 29)**	**a**
EL222-TF	HTH	>108	Transcription	~30 s	Motta-Mena et al., 2014
**VVD**	**Circadian clock**			**18000 s (WT)**	**a**
Caspase-9	Homo-dimerization	7.6–21	Caspase9 activation to regulate apoptosis	NA	Nihongaki et al., 2014
GAVPO	Gal4	200–300	Light induced transactivation of Gal4	7200 s	Chen et al., 2013; Ma et al., 2013
Magnets	Selective Dimerization of VVD	40-fold estimate	Create VVD heterodimers of two components	25 s–17000 h	Kawano et al., 2015
**FKF1**	**Flowering**			**>100000 s (WT)**	**a**
LITEZ	2-hybrid	53	Transcriptional control	NA	Polstein and Gersbach, 2012
LAD	Light induced dimerization	5	Rac1 induction of lamellipodia	62 h	Yazawa et al., 2009

a, refer to ***Table 2*** for more details on range of photocycle lifetimes.

## LOV photocycles and kinetics

All LOV proteins are defined by equivalent chemistry centered on the active site flavin and the Cysteine in the GXNCRFLQ motif (Salomon et al., 2000; Swartz et al., 2001; Crosson et al., 2003; Harper et al., 2003). Dark-state LOV proteins (ground state:LOV_450_) contain an oxidized flavin cofactor that maximally absorbs blue-light at 450 nm (Figure 1A). Upon blue-light absorption, LOV proteins rapidly form a covalent linkage between the C4a position of the flavin cofactor and the thiol moiety of the active site cysteine (Figure 1B). Although some debates remain in regards to the nature of reactive intermediates, a consensus mechanism can be described as outlined in Figure 1 (Holzer et al., 2002; Iwata et al., 2002; Bittl et al., 2003; Corchnoy et al., 2003; Kennis et al., 2003, 2004a; Schleicher et al., 2004; Dittrich et al., 2005; Sato et al., 2005; Alexandre et al., 2009a). Briefly, blue-light promotes LOV_450_ into a singlet-excited state that rapidly undergoes intersystem crossing. The triplet state then induces electron and proton transfer from the active site cysteine. Finally, the resulting radical species recombine to form the C4a adduct signaling state (S390) that is defined by a single broad absorption band centered at 390 nm. The photocycle is thermally reversible in the dark, decaying to the ground state on a timescale of seconds to days (see Table 2) (Zoltowski et al., 2009). The widely varying photocycle lifetimes have been of keen interest to researchers and their biological relevance is still weakly explored.

**Table 2:**
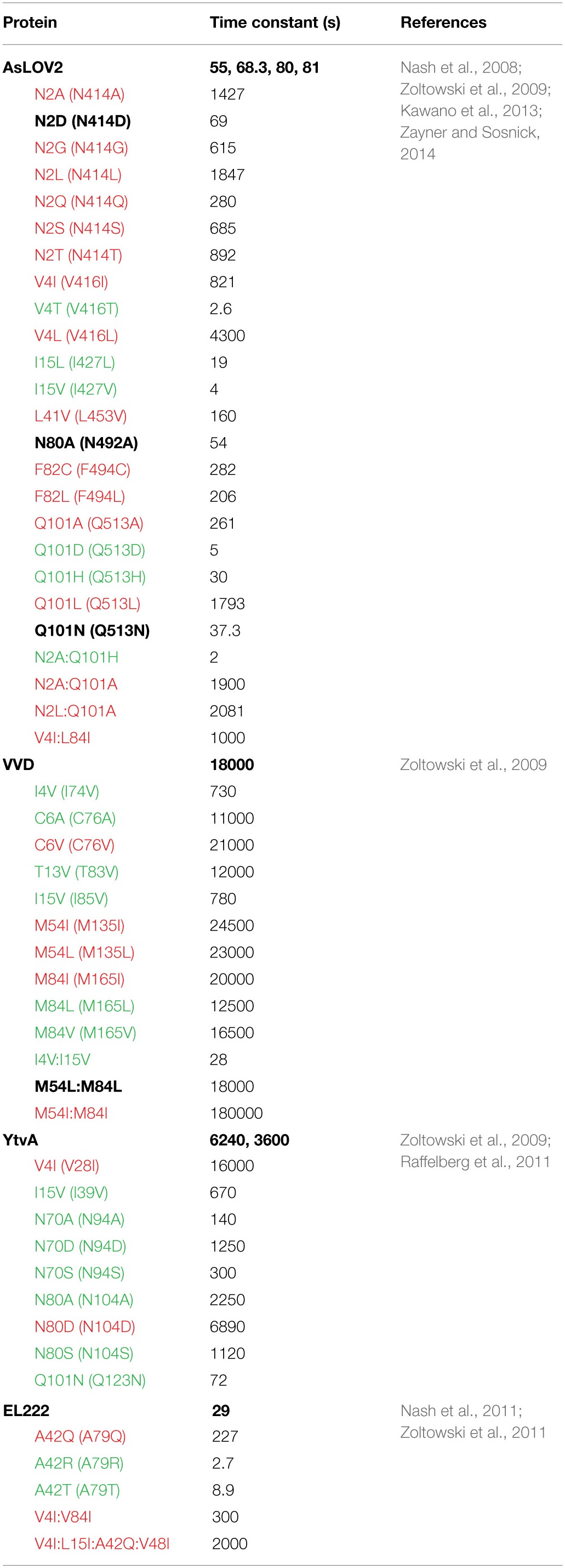
**Kinetics of thermal reversion for LOV constructs and variants at 296 K**.

Red: Slow mutations; Green: Fast mutations; Black: Small effect.

Currently, most research into the LOV photocycle centers on the large range in adduct decay kinetics. For the purpose of this review we will break down LOV photocycles as falling within three regimes: fast cycling (τ < 1000 s), intermediate cycling (1000 < τ < 10000 s), and slow cycling (10000 < τ s). Thus, existing optogenetic tools are either fast cycling (AsLOV2; ~80 s, EL222; ~30 s), intermediate (YtvA~6000 s) or slow cycling (VVD;~18000 s, FKF1; 100000 < τ. As noted below, the different photocycle lifetimes have significant impact on the sensitivity of optogenetic tools to different environmental light intensities as well as the dynamic reversibility of the systems (Zoltowski et al., 2009; Pudasaini and Zoltowski, 2013; Diensthuber et al., 2014).

Although, research into the *in vivo* effects of LOV photocycles is limited, recent studies of plant LOV photoreceptors indicate that the widely varying kinetics of adduct decay are important to dictating sensitivity to the intensity of environmental light (Okajima et al., 2012; Pudasaini and Zoltowski, 2013). Specifically, a UV-A light stimulated adduct decay pathway competes with blue-light activated formation of the C4a adduct (Kottke et al., 2003; Kennis et al., 2004b). These combine with thermal decay of the light-state species to generate a photodynamic equilibrium sensitive to environmental fluence (Pudasaini and Zoltowski, 2013). In this equilibrium, the rate of adduct decay specifies three regimes that differ in regards to their sensitivity to environmental light. The fast cycling LOV domains generate a dynamic equilibrium sensitive to all environmentally observed light-intensities. In contrast, intermediate LOV domains are completely saturated at moderate light intensities (greater than 20 μmole/m^2*^s), but retain peak sensitivity under low light conditions consistent with dusk/dawn (5–20 μmole/m^2*^s) (Pudasaini and Zoltowski, 2013). The third class of slow cycling LOV domains is exquisitely sensitive to even very low light intensities, where under natural lighting conditions the light/dark ratio is saturated. Although the biological relevance of these effects is still weakly explored, they have significant effects on the design of LOV based optogenetic tools. Namely, we are often forced into one of two regimes. Either we have a fast cycling LOV protein (AsLOV2/EL222) that requires high-intensity blue-light to saturate optogenetic signals, but affords rapid on/off kinetics, or one has a slow cycling protein (VVD/FKF1) that requires minimal light, but is limited in its on/off kinetics. For these reasons much research has gone into tuning these protein photocycles to afford a wide-ranging platform with diverse kinetic parameters.

## Tuning of LOV photochemistry lifetime

**Figure 2:**
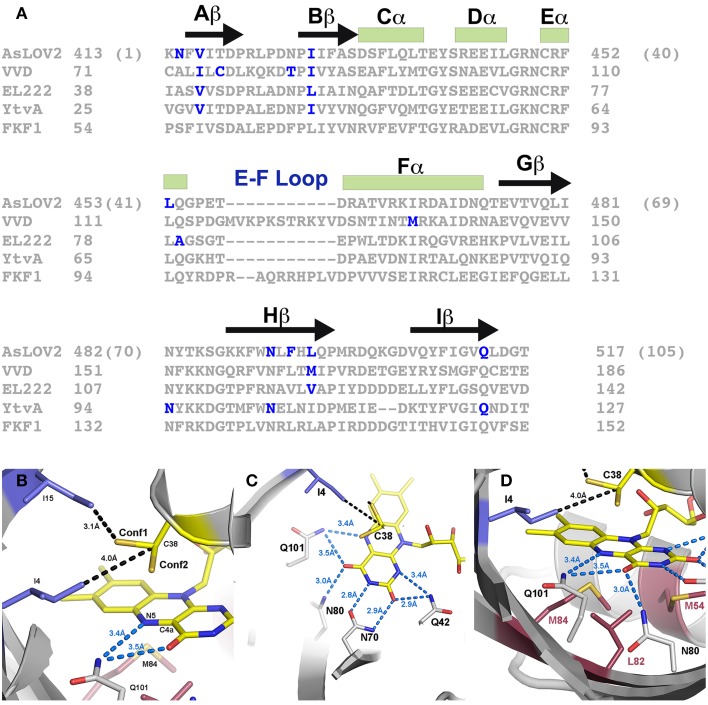
**Sites for rate altering variants. (A)** Sequence alignment and universal numbering scheme for LOV proteins and optogenetic tools. The numbering scheme (in parentheses for AsLOV2) used in this review references K413 of AsLOV2 as residue 1 of the core LOV domain. All residues are then numbered in reference to the alignment provided, where residue inserts (E-F loop) or deletions (YtvA) are ignored in the universal numbering system. Residues that have been targeted for rate altering effects are depicted in blue. **(B)** Steric interactions (blue residues) select for alternative conformations of C38. Conf2 places the thiol directly above the C4a position, where it is poised for C4a adduct formation. I4 juts in between the two conformations placing its methyl group only 4.0 Å away from Cβ. Rotation between the two conformations would require movement of I4. **(C)** A network of H-bonds in the pyrimidine ring stabilize the C4a adduct through electron withdrawing effects. **(D)** Full active site containing residues attenuating Conf1/2 (blue), residues at the re-face (red) and H-bonding residues (gray). Three residues, M54, L82, and M84 attenuate adduct decay pathways through steric and electronic regulation of the flavin.

Tuning of LOV photocycles has focused on three primary aspects of flavin chemistry and LOV structure (Christie et al., 2007; Nash et al., 2008; Zoltowski et al., 2009, 2011, 2013; Raffelberg et al., 2011; Song et al., 2011). First, dark-state LOV structures demonstrate two ground state conformations of the active site Cysteine (Fedorov et al., 2003; Christie et al., 2007; Sato et al., 2007; Zoltowski et al., 2009). Only one of these situates the Cysteine above the C4a position, where it is ideally poised for adduct formation (Figure 2B). Several studies have concluded that steric factors favoring an orientation away from the C4a position can destabilize the light-state adduct (Christie et al., 2007; Zoltowski et al., 2009; Kawano et al., 2013).

Second, adduct formation couples electron transfer and protonation of the N5 position of the isoalloxazine ring. These factors of LOV chemistry provide two practical means of attenuating adduct stability. (1) Factors that can stabilize increased electron density within the flavin system can stabilize the light state adduct. Thus, an increase in hydrogen bonding near the pyrimidine ring can stabilize the light-state species (Raffelberg et al., 2011; Zoltowski et al., 2011) (Figure 2C). (2) Factors that favor deprotonation of the N5 position of the isoalloxazine ring contribute to a faster decay pathway (Zoltowski et al., 2009, 2011; Raffelberg et al., 2011). This second factor is consistent with several reports indicating a single proton transfer event as being rate limiting in adduct decay (Corchnoy et al., 2003; Zoltowski et al., 2009, 2011; Pudasaini and Zoltowski, 2013). Further, adduct decay is readily base catalyzed by either increased solvent access to the active site, or the presence of exogenous bases such as imidazole (Kottke et al., 2003; Alexandre et al., 2007; Zoltowski et al., 2009, 2011; Purcell et al., 2010; Pudasaini and Zoltowski, 2013).

A third element of LOV photochemistry affecting adduct stability involves conformational changes within the isoalloxazine ring following adduct formation. The C4a adduct results in sp^3^ hybridization of the C4a position as well as a tilt in the planarity of the flavin ring (Zoltowski and Gardner, 2011). These alter local steric constraints, particularly in residues occupying a position directly below the isoalloxazine ring (re-face) (Figure 2D). In turn, these combine to enable the re-face of the flavin ring to attenuate adduct stability through alterations in steric and electronic properties of residues at these sites (Zoltowski et al., 2009). Below we focus on residues affecting each of these parameters in model optogenetic systems. Due to different numbering for amino acids in LOV proteins, multiple reports of the same amino acid affecting adduct decay rates exists. To highlight the equivalence of sites in affecting adduct decay, we devised a numbering system for the LOV core, where residue 1 is the first non-PAS core residue in LOV proteins (C71 in VVD; K413 in AsLOV2). Numbering is then based in relation to the most widely studied system, AsLOV2. An alignment of LOV proteins according to the universal system is provided in Figure 2A. To avoid complications caused by insertions or deletions, these regions are not included in the numbering, rather all residue numbers reflect the equivalent residue in AsLOV2 as shown in Figure 2A. In Table 2 we summarize rate-altering variants in four optogenetic systems, the corresponding residues in each protein as well as the generalized numbering system. Here forward we refer to residues by the generalized numbering system unless otherwise noted.

## Steric contacts at active site cysteine

The active site cysteine (C38) adopts two possible configurations within the active site (Fedorov et al., 2003; Kottke et al., 2006; Sato et al., 2007; Zoltowski et al., 2009). Conformation 1 (Conf1) orients the thiol group toward the dimethyl-benzene ring of the active site flavin and away from the C4a position (Figure 2B). Such a conformation is stabilized by interactions with ordered water at the terminal end of a conserved solvent channel. Conformation 2 (Conf2) involves rotation of the C38 side chain, positioning the reactive thiol directly above the C4a position. Computational studies indicate that rotation of C38 is required for adduct formation, leading to a higher quantum yield for Conf2 (Fedorov et al., 2003; Sato et al., 2007). Initial searches for residues affecting adduct decay in LOV proteins identified a residue directly above C38 that attenuated adduct decay by up to an order of magnitude (Christie et al., 2007). Such data led to a proposed model, whereby steric factors favoring Conf1, could promote accelerated adduct decay in LOV proteins (Christie et al., 2007).

An alternative mechanism based approach to tuning LOV photocycles further solidified a role of the C38 conformation in altering decay kinetics. Studies of the fungal photoreceptor VVD identified two residues contacting C38 that can select for Conf1/2 (Zoltowski et al., 2009). Consistent with a steric model of regulating LOV kinetics, isoleucine residues that sterically constrain C38 favor Conf2 and a stable adduct. In contrast, decreased sterics through valine variants favor Conf1 and acceleration in adduct decay (Zoltowski et al., 2009). Combined, these two studies identified two key residues that can alter adduct decay pathways in VVD, AsLOV2, EL222 and YtvA in a predicable manner, namely V/I4 and V/I15 (Zoltowski et al., 2009, 2013). However, these sites do not only affect the conformation of C38. The close proximity to a solvent channel also attenuates adduct decay through alteration of the stability of the N5 protonation state and H-bonding to the active site flavin (Christie et al., 2007; Zoltowski et al., 2009; Kawano et al., 2013).

## Hydrogen bonding and the N5 position

C4a adduct formation is coupled to an electron and proton transfer event. Detailed computational studies of LOV-type chemistry provide mechanistic details important to tuning LOV photocycle kinetics. A landmark approach by Domratcheva et al. calculated transition states for adduct formation and adduct scission (Domratcheva et al., 2006). These transition states include a significant build up of electron density on the N5 position, exhibiting a partial charge of −0.275, −0.327, and −0.204 in the transition states for adduct formation, the light-state adduct and the transition state for adduct decay, respectively (Domratcheva et al., 2006). Notably, the largest localization of charge on N5 occurs in the light-state adduct. Based on these calculations, any factors that can aid in delocalization of electrons in the isoalloxazine ring will contribute to tuning the reaction landscape in LOV proteins. Moreover, the most significant effect of electron withdrawing agents will occur in stabilization of the light state adduct, where the largest buildup of charge exists. Due to these factors, delocalization of electrons through electron-withdrawing effects of H-bonding residues near N1, O2, N3, and O4 can have a pronounced effect on adduct stability (Raffelberg et al., 2011; Zoltowski et al., 2011). Several studies have examined the effect of H-bonding residues on attenuation of LOV chemistry.

In YtvA, Raffelberg et al. performed a detailed analysis of H-bonding residues on LOV reaction dynamics (Raffelberg et al., 2011). Variants of residues N70 (H-bonding to O2 and N3) and N80 (H-bonding to O4) were shown to have a large effect on the spectral and kinetic properties of the LOV photocycle (Figure 2C). Consistent with the reaction mechanism calculated by Domratcheva et al. variants at these sites tuned the ground and excited state absorption profiles, altered the quantum yields of adduct formation, and tuned the activation energies and lifetimes of the light-state adduct. Combined they were able to tune the half life of adduct decay within the range of 72–7000 s (Raffelberg et al., 2011).

A similar approach identified another location where H-bonding residues can tune reaction dynamics within the LOV active site. Whereas, most proteins contain a H-bonding residue near the flavin N1 position, EL222 does not (Zoltowski et al., 2011). The lack of an H-bonding residue at this site (position 42) enables increased solvent access to the active site and alteration of the electronic properties of the flavin (Figure 2C). NMR studies confirm an increase in electron withdrawing effects at the N1 position through introduction of H-bonding residues. Consistent with the Domratcheva mechanism, these electron withdrawing effects correlate with an increase in adduct stability. Through a combination of steric variants (position 4 and 15) and H-bonding at the N1 position (position 42), Zoltowski et al. were able to tune the EL222 lifetime over a range of 3–2000 s (Zoltowski et al., 2011, 2013). Importantly, these studies indicated that one cannot fully separate the effects of H-bonding and solvent access to the active site in affecting LOV kinetics as they impinge on the rate limiting N5 deprotonation.

N5 deprotonation can either be achieved through spontaneous deprotonation, proton abstraction by an unknown endogenous base or external bases such as imidazole (Kottke et al., 2003; Alexandre et al., 2007). Several studies have concluded that base catalysis is attenuated by solvent access to the flavin active site through a conserved solvent channel (Zoltowski et al., 2009, 2011; Purcell et al., 2010; Pudasaini and Zoltowski, 2013). In all known LOV structures, ordered water is present adjacent to C38. FTIR and *in vivo* approaches conclude that these ordered water molecules contribute to the native decay pathway and that dehydration of LOV proteins leads to large effects on adduct decay kinetics (Chan and Bogomolni, 2012; Pennacchietti et al., 2014). Further, studies of the fungal photoreceptor VVD, a bacterial LOV histidine kinase LOVK and a short LOV (sLOV) protein identify two main factors affecting solvent access (Zoltowski et al., 2009, 2011; Purcell et al., 2010; Pudasaini and Zoltowski, 2013; El-Arab et al., 2015). These include two residues (I4 and I15) that sterically interact with Conf1 to occlude solvent access to the LOV active site (Zoltowski et al., 2009). In addition, Ncap and Ccap elements adjacent to the β-scaffold regulate solvent accessibility, presumably through stabilization of the LOV core (Purcell et al., 2010). Combined these sites can have up to a 1000-fold effect on solvent access as assayed by base catalysis efficiency (Zoltowski et al., 2009; Purcell et al., 2010; El-Arab et al., 2015).

## The flavin re-face

Initial research into LOV proteins focused on the LOV1 and LOV2 domains of phototropins. The LOV1 and LOV2 domains were distinguished by differences in their photocycle properties and structural dynamics. Specifically, LOV1 domains offer longer photocycle lifetimes and dampened conformational responses as measured by FTIR (Iwata et al., 2005; Yamamoto et al., 2008; Alexandre et al., 2009b). In contrast, LOV2 domains had fast cycling photocycles and FTIR analysis indicated large-scale disruption of the LOV β-sheet following photoactivation. Research into the source of these differences in LOV domain function identified a Phe→Leu substitution between LOV2 and LOV1 domains that impart altered conformational landscapes and photocycle kinetics. Specifically, a F1010L (position 82) variant directly beneath the isoalloxazine ring of Neo1-LOV2 led to a 10-fold slower photocycle lifetime (90 s vs. 870 s) and led to LOV1 type conformational dynamics (Yamamoto et al., 2008). These studies were the first to identify the re-face of the flavin ring system as a key region regulating LOV structure and dynamics.

The ability to tune LOV reaction dynamics through alterations of residues near the re-face of the flavin draws on mechanistic analysis of the LOV photocycle. Similar to electron withdrawing effects stabilizing a build-up of charge in the isoalloxazine ring, the re-face of flavins is sensitive to the local electronic environment. Specifically, studies of flavoproteins indicates that diffuse electron containing amino acids such as Phe and Met can contribute electron density to the isoalloxazine ring (Ghisla and Massey, 1989). In LOV proteins, such interactions would promote increased conformational dynamics following light activation and destabilize the build up of charge following C4a bond formation.

These properties of the flavin re-face were exploited in a later study focusing on naturally varying residues that distinguish LOV photocycle properties. The study identified a cluster of residues within the re-face that tune LOV function over several orders of magnitude (Zoltowski et al., 2009) (Figure 2D). In the fungal photoreceptor VVD, two Methionine residues alter the steric and electronic environment of the active site flavin to promote adduct decay (M54 and M84). Introduction of branched chain aliphatic residues (I/L) leads to a stabilization of electron density within the isoalloxazine ring. The stabilization is confirmed by a long-lived light-state adduct and stabilization of reduced semiquinone species (Zoltowski et al., 2009; Vaidya et al., 2011). These sites allowed extension of the C4a adduct lifetime to the order of days, allowing for the first direct determination of a light-state structure (Vaidya et al., 2011). Combined, the studies indicate that the re-face can contribute to LOV kinetics and signaling through two interlocked manners. First, diffuse electron containing amino acids (M/F) destabilize the light state adduct and amplify conformational changes. In contrast, branched chain aliphatic residues promote steric constraints and charge stabilization on the active site flavin, thereby dampening conformational changes and promoting a stable light-state adduct.

## Exploitation of the LOV photocycle in optogenetic tools

Research into the divergent photocycle lifetimes of LOV proteins enables tuning of LOV kinetics by over four-orders of magnitude. These offer great potential in affording a tunable platform for optogenetic tools; however, exploitation of LOV photocycle properties in optogenetics has been fairly limited. Here we discuss some useful applications that result from altering LOV photocycle properties. In addition, we demonstrate current limitations to the above approach to alter LOV photocycle kinetics for tunable optogenetic tools.

Currently, two categories of optogenetic tools directly exploit properties of the LOV photocycle for an engineered cell biology tool. Both take advantage of fluorescent properties of LOV proteins to either develop new fluorescent imaging tools (iLOV, BsFbFP, and PpFbFP) or for possible implementation in super-resolution microscopy (Drepper et al., 2007; Chapman et al., 2008). A recent review of LOV proteins as fluorescent reporters provides detailed commentary on their development and improvement (Mukherjee and Schroeder, 2015), here we provide a brief synopsis of fluorescent LOV reporters and their utility. iLOV, BsFbFP, and PpFbFP take advantage of the fluorescent properties of dark-state LOV proteins (AsLOV2, YtvA, and a LOV protein from *Pseudomonas putida*, respectively) to allow the development of an oxygen independent fluorescent reporter (Drepper et al., 2007; Chapman et al., 2008; Gawthorne et al., 2012; Wingen et al., 2014). Initial work in fluorescent LOV reporters was conducted by Drepper et al. where they demonstrated oxygen-independent activity that for BsFbFP and PpFbFP that allowed anaerobic imaging. All these systems rely on swapping C38, required for adduct formation, with an inactive alanine to improve fluorescent properties of LOV proteins. Subsequent work by Chapman et al. used directed evolution approaches to improve the fluorescent properties of the iLOV system. The results of these studies were the development of a robust alternative to GFP reporter systems (Drepper et al., 2007; Chapman et al., 2008; Mukherjee and Schroeder, 2015). Additional studies have greatly improved the quality of fluorescent LOV reporters and extended their utility to additional approaches (i.e., metal sensing) (Drepper et al., 2010; Christie et al., 2012b; Ravikumar et al., 2014). These fluorescent LOV reporter systems exhibit brightness competitive with GFP, but with improved stability (reversible photobleaching) and functionality in low-oxygen or anaerobic conditions.

The second exploits both the UV-catalyzed adduct decay pathway that results in a steady-state light/dark photostationary state, and the fluorescent properties of dark-state LOV proteins. Specifically, researchers identified that violet/UV light can promote adduct scission, resulting in a photoswitchable fluorescent reporter. They proposed that these systems can be exploited in YtvA for super-resolution microscopy approaches (Losi et al., 2013). The photoswitchable fluorescent properties make LOV proteins a possible template for fluorescence photoactivation localization microscopy (FPALM), however these systems are still currently being optimized for improved performance in cellular systems. Several factors, including the lifetime of the light-state adduct and differences in *in vitro* and *in vivo* photochemical properties limit these approaches (Pennacchietti et al., 2014).

Unfortunately initial attempts to incorporate rate-altering variants into optogenetic tool design have been hampered by unexpected effects on signal propagation. Rate altering variants have only been used in VVD (Wang et al., 2012; Chen et al., 2013; Ma et al., 2013; Kawano et al., 2015), and AsLOV2 (Strickland et al., 2008) platforms. Examination of variants in these and other systems indicates that they can often grossly affect signal propagation, thereby damaging the fidelity of the optogenetic tools (Gleichmann et al., 2013; Kawano et al., 2015). Specifically, a large random mutagenesis approach aimed at examining residue substitutions in an optogenetic device revealed that many of the sites targeted for affecting LOV photocycle lifetimes have deleterious effects on signal propagation (Gleichmann et al., 2013). These deleterious effects were significant in all variants that disrupt H-bonding contacts to the active site flavin (e.g., N70, N80, Q101). Only aliphatic sites showed minimal effect on signal propagation. Thus, in order to have greater control of optogenetic tools, we are forced to both consider chemical parameters and their structural consequences. Therefore, it is of keen interest to understand signal propagation in LOV systems to provide tunable LOV optogenetic devices.

## Signal transduction mechanisms

**Figure 3:**
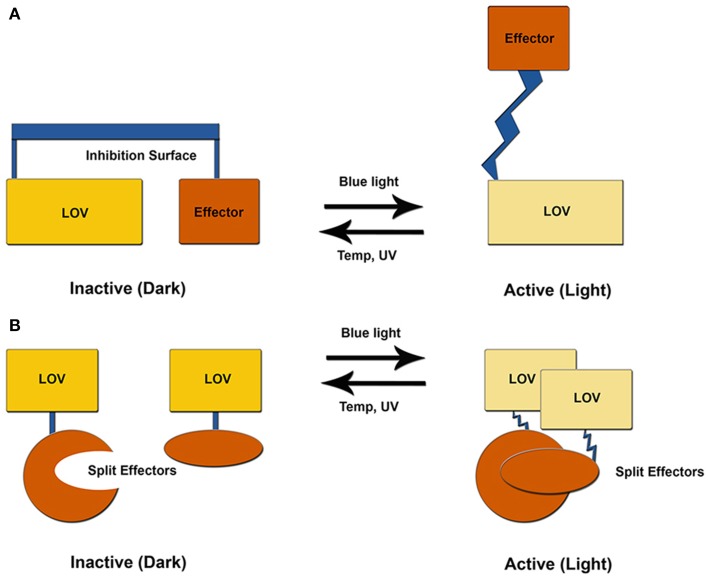
**LOV optogenetic tools**. Existing LOV-based tools exploit one of two possible mechanisms. **(A)** LOV and effector are attached through a helical linker to create an inhibitory surface that is released following photoexcitation. **(B)** An effector molecule is split into two inactive components. Light activation induces LOV-mediated dimer formation to activate the effector molecule.

LOV structures are distinguished by three general factors. All photoreactive elements are confined to a core PAS domain defined by the central β-scaffold and a helical interface that house the photoreactive flavin (Zoltowski and Gardner, 2011). Signal propagation, however, is isolated to Ncap and Ccap extensions to the PAS core that afford the capacity to inhibit signal transduction through sequestration or constrainment of a signaling motif (Figure 3) (Crosson et al., 2003; Halavaty and Moffat, 2007; Zoltowski et al., 2007; Zoltowski and Gardner, 2011; Diensthuber et al., 2013). These elements can exist alone in short LOV proteins (sLOV) or as a linker to signal transduction domains (e.g., histidine kinase, F-box, GAF domain, GGDEF domain etc…). Downstream signaling then focuses on several allosteric mechanisms of signal transduction stemming from C4a adduct formation and protonation of the N5 position. While initially hoped to function as a light switch between inactive (dark) and active (light) states, all characterized systems exist as more of a “dimmer-model,” where all proteins retain some dark-state function that can be amplified by increasing light-intensities (Crosson and Moffat, 2001; Strickland et al., 2008, 2010; Pudasaini and Zoltowski, 2013). These aspects currently limit the fidelity of LOV optogenetic tools (Table 1). We begin by recapping key elements of the LOV photocycle that initiate signal transduction before focusing on mechanisms for each of the model optogenetic systems below.

**Figure 4:**
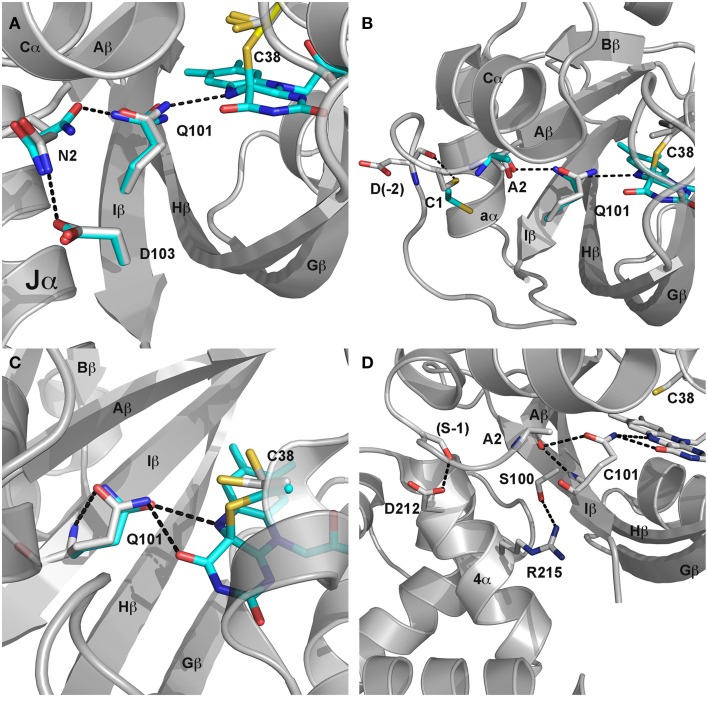
**LOV signaling mechanisms. (A)** Dark (gray) and light (cyan) state structures of AsLOV2. Photoactivation leads to rotation of Q101 to alter H-bonding contacts to N2. N2 undergoes a light driven interchange involving contacts with D103 and Q101. The H-bond switch affects Ncap structure to disorder the Jα helix. **(B)** Dark (gray) and light (cyan) state structures of VVD. C4a adduct formation promotes rotation of Q101 to alter H-bonds to A2. Movement of the Ncap reorientates C1 to disrupt contacts with D-2, leading to rearrangement of the Ncap and dimer formation. **(C)** (YtvA) and **(D)** (EL222) mechanisms are less understood but likely involve Q101 and H-bonding contacts (black dotted line) to neighboring residues.

Mechanistic studies of the LOV photocycle by numerous researchers identify two chemical elements that can initiate signal transduction in LOV systems. As noted above, these are inherently coupled to approaches to tune reaction dynamics and kinetics. First, adduct formation results in protonation of the flavin N5 position. N5 protonation in turn alters the H-bonding landscape near the flavin active site that has the capacity to induce allosteric conformational responses (Halavaty and Moffat, 2007; Zoltowski et al., 2007; Freddolino et al., 2013). Second, adduct formation results in a build up of electron density within the isoalloxazine ring, primarily centered on the N5 and C4a positions (Domratcheva et al., 2006). The build up of charge can be read out by nearby diffuse electron containing amino acids (F/M) that typically occupy positions near the re-face. As detailed by FTIR studies differentiating LOV1 and LOV2 domains of phototropins these residues can propagate conformational changes through disruption of the LOV β-sheet (Iwata et al., 2003, 2005; Yamamoto et al., 2008; Alexandre et al., 2009b). These aspects indicate that conformational changes initially propagate from residues within Aβ, Iβ and N/Ccap elements as detailed below (Figure 4).

## AsLOV2

The LOV2 domains of plant phototropins are the most heavily studied of all LOV domains. Similarly, the AsLOV2 domain is the most commonly exploited LOV protein for optogenetic tools. Despite extensive characterization, the development of new robust tools based on an AsLOV2 platform remains challenging. These in part stem from residual dark-state activity of AsLOV2 proteins that limit the fold-amplification of light state signals (Table 1). We outline the current models of signal propagation in this system here, with specific emphasis on methods used to optimize LOV2 signaling in optogenetic tools.

Similar to many of the LOV domains, signal propagation in AsLOV2 is initiated by a combination of C4a adduct formation and N5 protonation (Figure 4A). Protonation of the N5 position alters H-bonding contacts to a glutamine residue (Q101) conserved in the majority of LOV proteins. FTIR, NMR, crystallographic and computational studies identify Q101 as the locus for signal transduction, albeit with slight deviations in the mechanisms of signal propagation (Harper et al., 2003, 2004; Iwata et al., 2003, 2005; Nozaki et al., 2004; Freddolino et al., 2006, 2013; Halavaty and Moffat, 2007; Nash et al., 2008; Yamamoto et al., 2008; Alexandre et al., 2009b). All models couple adduct formation to a marked reduction in the helical content of LOV2 proteins as well as a reduction in β-sheet contacts. These led to a model of signal propagation centered on the Hβ and Iβ (contains Q101) strands that allosterically regulate a C-terminal helix (Ccap; Jα). NMR studies indeed confirmed that adduct formation results in a weakening of the β-scaffold and light-driven unfolding of Jα (Harper et al., 2003, 2004).

Recent crystallographic structures of extended AsLOV2 structures indicate that the Ccap is not the only locus for structural regulation of effector proteins. Dark-state structures and dark-grown crystals exposed to blue-light provided a mechanism of signal propagation linking the flavin active site to both the Ncap and Ccap. N-terminally extended structures revealed an additional short helical element directly preceding the Aβ strand. The Ncap element also directly interacts with Jα, thereby linking Ncap and Ccap signaling elements (Halavaty and Moffat, 2007). These two helical elements are connected to the N5 position by Q101, which undergoes a light-driven switch following adduct formation. Briefly, the N5 proton alters H-bonding interactions between the flavin, Q101 and N2 in Aβ (Halavaty and Moffat, 2007). In light of previous NMR data, indicating unfolding of Jα, a consensus model of allosteric regulation was obtained. In the consensus model adduct formation alters H-bonds to Iβ and Aβ to favor dislodgement and unfolding of Jα. These in turn relieve inhibitory contacts between the LOV core and downstream signaling elements (Figure 4A).

At present, optogenetic tools exploit light-driven unfolding of a Ccap helical element to regulate an effector module through one of two possible pathways. These often rely on linking an effector protein through a compound helix coupled to Jα. Resulting unfolding of the Jα helix can then be used to relieve inhibition of the effector module through release of steric constraints, or to expose the Jα helix for light-driven interactions with an effector protein (Figure 3). These systems are best demonstrated by a LOV-Rac fusion (PA-Rac1) and a LOV based modification to the mammalian two-hybrid system, although several other platforms exist (Table 1) (Strickland et al., 2008, 2010; Wu et al., 2009; Lungu et al., 2012).

In PA-Rac1, the AsLOV2 domain is fused to Rac through a C-terminal compound helix composed of the Jα helix and N-terminus of Rac (Wu et al., 2009). Light activation leads to rotation of Q101, to unfold the compound helix, relieving steric constraints at an inhibitory surface between the LOV helical face and Rac. Although, PA-Rac1 is a robust optogenetic tool, crystallographic structures of the fusion protein highlight several complications in designing composite optogenetic devices. Namely, design of the compound helix must retain sufficient elements to maintain LOV-type signaling, but allow for close retention of the effector domain to develop an inhibition surface.

An alternative approach to AsLOV2 optogenetic tools is to couple allosteric regulation of the Jα helix to induce light driven dimerization. Two research groups have exploited this approach to induce gene transcription through modified yeast/mammalian 2-hybrid approaches or cellular colocalization. These systems attach a peptide-recognition element to the C-terminal end of the Jα helix. In the dark-state, binding of the Jα helix to the LOV core constrains the peptide recognition element rendering it incapable of binding to its cognate effector domain. Light activation disorders the Jα helix relieving constraints and leading to protein:protein interactions. Although conceptually based on the same principle, the approaches differ in their recognition element and cognate effector.

The first system, termed TULIPS (Tunable, Light-controlled Interaction Proteins), involved fusing a peptide designed to interact with PDZ domains (Strickland et al., 2012). These systems demonstrated remarkable ability to direct cellular localization of target proteins. Building upon previous attempts to optimize the fidelity of the light-dark switch (Strickland et al., 2010), Strickland et al. devised an optimal construct by coupling variants within the Ncap and Ccap helices to repress residual dark-state activity. A combined T406A, T407A (Ncap) and I532A (Ccap) (AsLOV2 numbering) triple variant abolished dark-state activity, but retained light-dark switching (Strickland et al., 2012). Further, they directly employed rate-altering variants (V4I) to demonstrate that *in vitro* approaches to alter LOV kinetics translated to *in vivo* function.

Kuhlman, Hahn and coworkers used an analogous approach, where they caged peptide elements recognized by Vinculin as modified Jα helices (ipaA and SsrA) (Lungu et al., 2012; Yi et al., 2014). Using molecular modeling they designed chimeric Jα helices that retained elements required for LOV docking and Vinculin binding. The resulting chimera protein demonstrated high affinity in the light and dark with 19-fold amplification in binding following blue-light exposure. Rational design of protein variants to repress dark-state binding identified two residues L514K and L531E (AsLOV2 numbering) that decrease dark-state binding, while having only minor effects on the light-state. The resulting system demonstrates a robust 49-fold amplification in affinity following light-treatment. A more recent study aimed at improving the SsrA system identified key structural elements that allow tuning of the light-dark binding affinity of LOV-SsrA and its cognate receptor (Guntas et al., 2015). A key highlight of the two approaches is that residues that attenuate signal amplification (L531E/I532A) do not necessarily apply to all systems, but rather key elements within the chimera proteins distinguish the conformational landscape.

## VVD

VVD is a sLOV protein from *Neurospora crassa* involved in adaptation to increasing levels of blue light (Schwerdtfeger and Linden, 2003; Elvin et al., 2005). VVD contains only the photoactive LOV domain fused to an Ncap required for signal transduction (Zoltowski et al., 2007). In contrast to AsLOV2, signal transduction in VVD does not require an effector domain, rather involves competitive light-driven formation of protein:protein complexes (Zoltowski and Crane, 2008). Structurally, VVD is one of only two LOV containing proteins that have been crystalized directly as dark-state and light-state proteins offering keen insight into signal propagation in sLOV proteins.

Initial crystal structures revealed a mechanism of signal transduction closely related to AsLOV2, but differing in its functional output. Briefly, direct rotation of Q101 was observed following N5 protonation (Zoltowski et al., 2007; Vaidya et al., 2011). Rotation of Q101 to favor H-bonds to the newly protonated N5 propagates out to the surface through interactions with A2 (position 72 VVD numbering) within Aβ (Figure 4B). These in turn lead to rearrangement of Ncap elements through a conserved hinge region (Zoltowski et al., 2007; Lamb et al., 2008, 2009; Vaidya et al., 2011; Lokhandwala et al., 2015). Essential to signal propagation is C1, which rotates from a buried position between the LOV core and Ncap. These movements expose a hydrophobic cleft to support homodimer formation through reorganization of Ncap elements. SAXS and light-state crystal structures confirm reorganization of the Ncap to favor a low affinity, rapidly dissociating dimer.

Light-driven dimer formation ideally suits VVD for optogenetic control of protein:protein interaction, however the low dimer affinity and long lifetime limit its fidelity. Several approaches have been used to both employ and optimize VVD as an optogenetic tool. An early approach was the VVD light-on system, which is a modification of yeast and mammalian 2-hybrid approaches (Chen et al., 2013; Ma et al., 2013). Several attempts to optimize the light-on system have been conducted that exploit photochemical and structural mechanisms of signal transduction (Ma et al., 2013). A more recent approach termed magnets, has further evolved the VVD system to maximize light-amplification of signal with desired on/off kinetics (Kawano et al., 2015). Since the two approaches are similar we focus on the more recent magnets system to highlight structure and chemical tuning of optogenetic tools.

The VVD-based magnet system focused on alleviating two limitations of VVD-based tools, namely slow on/off kinetics and weak dimerization. The magnet system examined the key Ncap signaling region to evolve a pair of VVD variants capable of hetero-dimerization, but incapable of homodimerization. Specifically, they introduced charged residues at key dimer contact regions (Ile52 and Met55; VVD numbering). By creating a VVD pair with negatively charged resides at Ile52/Met55 and a positively charged version, Kawano et al. were able to design a robust system with ~40-fold activation upon light treatment (Kawano et al., 2015). By incorporating slow cycling variants in one component, paired with fast cycling elements in the other species, they were further able to amplify signal output and introduce improved on/off kinetics. The resulting system has not been employed widely, but affords tunable kinetics (four-orders of magnitude) and signal output (up to 1300% increase in signal intensity) (Kawano et al., 2015).

## EL222

EL222 was initially discovered as one of four LOV domain containing signaling proteins in the marine bacterium *E. litoralis* (Swartz et al., 2007). The 222-amino acid protein contains an N-terminal LOV domain directly coupled to an HTH-DNA binding domain through a short C-terminal linker (Nash et al., 2011). The HTH domain acts in a manner analogous to Ncap and Ccap elements in VVD/AsLOV2, where the α4 dimerization helix of the HTH domain docks to the LOV β-scaffold. NMR and crystallographic studies confirm a signaling mechanism that incorporates both Ccap reorientation and dimerization (Nash et al., 2011; Zoltowski et al., 2013).

Adduct formation is believed to propagate to the C-terminal HTH domain through rotation of Q101 that leads to unfolding of the C-terminal linker and release of the α4 helix of the HTH domain (Nash et al., 2011) (Figure 4D). Release of steric constraints on the HTH domain facilitates dimerization of EL222 through both the N-terminal LOV domain and α4 (Zoltowski et al., 2013). EL222 dimerization is also facilitated by DNA binding to two copies of a 5 bp RGNCY consensus motif separated by 2 A/T base pairs (Y = C/T, R = A/G, N = any nucleotide) (Rivera-Cancel et al., 2012). DNA binding in turn can be harnessed for activation of gene transcription using methods analogous to VVD and AsLOV2 above (Motta-Mena et al., 2014).

Several attempts to optimize EL222 function through both chemical and structural tuning have been employed. Mechanism based tuning EL222 chemistry currently allow for lifetimes between 2 and 2000 s (Zoltowski et al., 2011, 2013). These variants have not been tested for fidelity in optogenetic tool function, however they reversibly bind DNA following light-dark cycles (Zoltowski et al., 2013). Further, signal propagation has been optimized through identification of a high affinity DNA binding site through both Chip-seq and SELEX approaches (Rivera-Cancel et al., 2012). Current iterations of the EL222 system afford over 100-fold signal amplification with rapid on/off kinetics (Motta-Mena et al., 2014). Mathematical modeling of DNA binding and photocycle properties has identified a substantial role of the LOV lifetime in dictating temporal control of gene transcription (Motta-Mena et al., 2014).

## YtvA

YtvA regulates light-activated stress response pathways in *B. subtilis*. It is the best-characterized bacterial LOV protein, affording detailed knowledge of structural and chemical regulation of signaling mechanisms (Bednarz et al., 2004; Buttani et al., 2007; Avila-Perez et al., 2009; Mansurova et al., 2011; Raffelberg et al., 2011; Engelhard et al., 2013; Losi et al., 2013). These detailed studies enable widespread usage in optogenetic tools. Currently, these employ a general mechanism that bares homology to those present in AsLOV2. Namely, primary signaling mechanisms proceed through C4a adduct formation to regulate a C-terminal effector domain through allosteric regulation of a Jα helix (Losi et al., 2005; Buttani et al., 2007; Engelhard et al., 2013) (Figure 4C). However, in contrast to AsLOV2, where unfolding of Jα mediates signaling, YtvA structural studies suggest signal propagation results from alteration of the Jα helical tilt (Tang et al., 2010; Engelhard et al., 2013). The helical tilt has been harnessed to regulate C-terminal effectors to regulate histidine kinases and gene transcription (Moglich et al., 2009; Ohlendorf et al., 2012; Diensthuber et al., 2013; Gleichmann et al., 2013).

The initial YtvA system was constructed by swapping the YtvA LOV domain, including the Jα helix with the PAS regulatory domain of the histidine kinase FixL (YF1). The resulting system imparted light-regulated control of kinase activity (Moglich et al., 2009). Through alteration of the length of the Jα-FixL helical linker, Möglich et al. were able to tune functionality to enable either light-state or dark-state kinase activity. In a novel extension to the YF1 system, they exploited the transcription activity of the cognate response regulator FixJ to enable light-activate gene regulation in bacterial species (Ohlendorf et al., 2012). The resulting Dusk/Dawn system allows for both light-activation and light-repression of gene transcription depending on the YF1 system employed. In both systems up to 460-fold induction of gene transcription is possible (Ohlendorf et al., 2012).

Several iterations of the YF1 system have been developed that optimize signal transduction as well as photochemical properties. Structural studies of a full-length YF1 chimera indicate that signal transduction impinges on both Ncap and Ccap elements, which undergo an alteration in helical pitch at N/C-terminal coiled-coil dimerization helices (Engelhard et al., 2013). These are coupled to LOV domain photochemistry through multiple H-bonding interactions. Mutational analysis of residues propagating signal transduction can tune the output and directionality of the Dusk/Dawn system. An analogous study examined the effect of residues lining the flavin active site (Gleichmann et al., 2013; Diensthuber et al., 2014). These indicated that residues employed to tune LOV photocycles could have deleterious effects on signal propagation, indicating that in some cases chemical and structural tuning cannot be separated.

## FKF1

The plant photoreceptor FKF1 contains a single LOV domain, which binds to Gigantea (GI) following blue-light activation (Imaizumi et al., 2003; Sawa et al., 2007; Baudry et al., 2010). Biological studies indicate that only the N-terminal LOV domain of FKF1 is necessary for light-induced dimerization with GI (Sawa et al., 2007). Currently, two systems exploit the light driven FKF1-LOV:GI interaction for optogenetic tools (Yazawa et al., 2009; Polstein and Gersbach, 2012).

A recently reported system LITEZ utilizes blue light induced interaction between FKF1-LOV and GI to induce gene expression (Polstein and Gersbach, 2012). The design of this optogenetic tool resembles a 2-hybrid gene expression system, where one component (GI) binds DNA through inclusion of an N-terminal Zinc Finger domain. The photoreactive FKF1-LOV then activates gene transcription through recruitment of a C-terminal VP16 element to GI following light-activated LOV:GI complex formation.

The LITEZ system has been reported to be very efficient with up to 53-fold increase in gene activation (luciferase) following blue light treatment (Polstein and Gersbach, 2012). Due to the long-lived photocycle of FKF1-LOV (100000 s < τ), it is not necessary to continuously illuminate the live cells. However, the photocycle half-life of FKF1-LOV currently limits the system in regards to on/off kinetics. Due to a lack of detailed studies of FKF1 structure and kinetics, the system is the least characterized and is limited in the ability to fine tune signal amplification and on/off kinetics.

## Concluding remarks

LOV proteins afford a unique platform for coupling blue-light activation to a wide range of signal transduction elements. Although significant detail is known for chemical and structural mechanisms in these systems, there still remains a limitation to the design and fidelity of LOV-based tools. Further research into light-state crystal structures of LOV proteins as well as LOV optogenetic tools is needed to enable improved, robust design of optogenetic devices. Several key areas are noted here for future development.

Currently, structural studies of LOV proteins are, in most cases, limited to the isolated LOV domains. Few structures exist for full-length or multi-domain containing LOV proteins. These limit our understanding of allosteric mechanisms in LOV containing proteins and similarly optogenetic tools. Structural studies of LOV domains with extended N- and C-terminal regions indicate that these elements are essential to light-dark switching, even without the downstream effector domains. Subsequent studies have shown that targeting these elements for mutagenesis is a robust method for tuning optogenetic function. To further extend the utility of these tools it is essential for researchers to better understand the natural mechanisms coupling LOV dynamics to downstream effectors through the N- and C-terminal linkages.

Extension of our understanding of allosteric mechanisms of signal transduction in LOV proteins and optogenetic tools should leverage existing efforts to tune LOV domain chemistry. Direct determination of light-state structures has been facilitated by rate-altering variants. These structures afford snapshots of the light and dark-adapted states that facilitate understanding of optogenetic tools. Unfortunately, few studies have examined the effects of these variants on *in vivo* function or optogenetic tool utility. Going forward, it is recommended that studies of rate altering variants and corresponding light state structures be conducted in concert with their effect on *in vivo* function and optogenetic tool design. Such efforts may provide a global understanding of how LOV chemistry and structure regulate signal transduction and allostery.

### Conflict of interest statement

The authors declare that the research was conducted in the absence of any commercial or financial relationships that could be construed as a potential conflict of interest.
